# The use of Cincinnati prehospital stroke scale during telephone dispatch interview increases the accuracy in identifying stroke and transient ischemic attack symptoms

**DOI:** 10.1186/1472-6963-13-513

**Published:** 2013-12-11

**Authors:** Assunta De Luca, Paolo Giorgi Rossi, Guido Francesco Villa

**Affiliations:** 1 Health Direction of Regional Authority of Emergency Services (ARES 118) Lazio Region Italy. New affiliation, Health Direction of Sant’Andrea Hospital Sapienza Rome University, Via Tronto 32, Roma, CAP 00198 Italy; 2 Servizio interaziendale di epidemiologia AUSL, Reggio Emilia, Italy; 3 Pre hospital emergency Operative Center of Lecco and coordinator of Italian Group Pre-hospital management of acute stroke – Italian Society of pre hospital emergency Services (SIS118). New affiliation: Azienda Regionale Emergenza Urgenza (AREU), Milan Lombardy, Italy

**Keywords:** Cincinnati Prehospital stroke scale, Emergency medical system, Stroke, TIA, Stroke and/or TIA symptoms

## Abstract

**Background:**

Timely and appropriate hospital treatment of acute cerebrovascular diseases (stroke and Transient Ischemic Attacks - TIA) improves patient outcomes. Emergency Medical Service (EMS) dispatchers who can identify cerebrovascular disease symptoms during telephone requests for emergency service also contribute to these improved outcomes. The Italian Ministry of Health issued guidelines on the management of AC patients in pre-hospital emergency service, including Cincinnati Prehospital Stroke Scale (CPSS) use.

We measured the sensitivity and Positive Predictive Value (PPV) of EMS dispatchers’ ability to recognize stroke/TIA symptoms and evaluated whether the CPSS improves accuracy.

**Methods:**

A cross-sectional multicentre study was conducted to collect data from 38 Italian emergency operative centres on all cases identified with stroke/TIA symptoms at the time of dispatch and all cases with stroke/TIA symptoms identified on the scene by the ambulance personnel from November 2010 to May 2011.

**Results:**

The study included 21760 cases: 18231 with stroke/TIA symptoms at dispatch and 9791 with symptoms confirmed on the scene. The PPV of the dispatch stroke/TIA symptoms identification was 34.3% (95% CI 33.7-35.0; 6262/18231) and the sensitivity was 64.0% (95% CI 63.0-64.9; 6262/9791). Centres using CPSS more often (>10% of cases) had both higher PPV (56%; CI 95% 57–60 vs 18%; CI 95% 17–19) and higher sensitivity (71%; CI 95% 87–89 vs 52%; CI 95% 51–54).

In the multivariate regression a centre’s CPSS use was associated with PPV (beta 0.48 p = 0.014) and negatively associated with sensitivity (beta -0.36; p = 0.063); centre sensitivity was associated with CPSS (beta 0.32; p = 0.002), adjusting for PPV.

**Conclusions:**

Centres that use CPSS more frequently during phone dispatch showed greater agreement with on-the-scene prehospital assessments, both in correctly identifying more cases with stroke/TIA symptoms and in giving fewer false positives for non-stroke/TIA cases. Our study shows an extreme variability in the performance among OCs, highlighting that form many centres there is room for improvement in both sensitivity and positive predictive value of the dispatch. Our results should be used for benchmarking proposals in the effort to identify best practices across the country.

## Background

Timely and appropriate hospital treatment of non-traumatic acute cerebrovascular diseases (AC) improves patients’ outcomes. For this reason, stroke is considered one of the Quintet of life-threatening emergencies (First Hour Quintet) [1]. Evidence confirms that despite technological advances, early and accurate clinical assessment remains the primary method for identifying patients with either stroke or transient ischemic attacks (TIA) [2-7].

The three main factors that may reduce pre-hospital delay are the patient’s or witness’s prompt identification of stroke signs and TIAs and immediately calling the Prehospital Emergency Medical Service (EMS), the rapid recognition of stroke symptoms by EMS dispatchers, and organized, timely, and efficient transportation towards appropriate facilities by EMS ambulances [8-12].

The timeliness and quality of care provided by the EMS significantly affect the outcome of patients with cerebrovascular diseases. These are the main reasons why the EMS must optimize response to stroke/TIA calls.

When patients with AC symptoms or their proxies call the EMS, the dispatcher, i.e., the first person in the emergency chain, should be able to recognize the symptoms of stroke/TIA. The dispatcher must then immediately establish the response priority for these calls as accurately as possible. EMS dispatchers have varying levels of accuracy in stroke/TIA recognition. Most EMS operators follow standardized protocols for phone interviews to identify the medical condition of the callers. These protocols often include an algorithm for cerebrovascular accidents [13-15]. There is evidence that the use of standardized methods (e.g., stroke scales, structured questionnaires) by EMS personnel (dispatchers and paramedics on the scene) to detect patients with suspected stroke/TIA improves identification rapidity and accuracy [16-24].

The tool that is most frequently mentioned in the literature and that helps EMS personnel to accurately identify stroke/TIA symptoms is the Cincinnati Prehospital Stroke Scale (CPSS) [25,26]. While many studies on CPSS reproducibility involve on-the-scene EMS healthcare professionals or laypersons [27-30], a few recent studies on CPSS have focused on EMS dispatchers [18,23].

In Italy, the emergency medical system (Prehospital Emergency Medical Service and hospital emergency departments) is assigned to 21 regional authorities. The Prehospital Emergency Medical Service (EMS), activated by dialing 118 around the clock, is regulated by national legislation and receives public funding. The organization of EMS calls and dispatch, however, varies from region to region: operative centers – OCs – answer to Local Health Authorities or to the Regional Authority of Emergency Services. The EMS dispatchers are nurses, and a physician supervises the dispatch center and evaluates critical situations where medical support is needed. In general, dispatch priority is based on a standardized questionnaire. The healthcare provided ranges from Basic Life Support by nurses and/or volunteers to full Advanced Life Support by emergency physicians on ambulances and/or helicopters. In general, the OCs transport patients to emergency departments (EDs) located in the same district.

While Italian national and regional guidelines all recommend that paramedics, nurses, and doctors use CPSS to identify AC signs and symptoms on the scene or in the ED [31], dispatch protocols for stroke/TIA symptoms identification are different throughout the 101 OCs nationwide, and only some have adopted CPSS. The majority of Italian regions have stroke centers [31], some of which are connected with the EMS; in this case patients with acute stroke/TIA symptoms are identified on the scene and subsequently transported to the nearest stroke center.

### Objective

Since the management of acute stroke/TIAs by EMS service differs from one Italian region to another, at the end of 2010, the Italian Society for the prehospital emergency Services 118 (SIS118) started a six-month cross-sectional study to record the patients with acute stroke/TIA symptoms that had contacted the EMS. In this paper, we analyze the accuracy of dispatchers’ recognizing stroke/TIA symptoms in terms of sensitivity and positive predictive value (PPV) using symptoms identification by pre-hospital assessment on the scene as reference. We also assess how the use of CPSS affects dispatchers’ accuracy in identifying stroke/TIA symptoms (at the operative center level).

## Methods

### Setting

All 101 EMS OCs in Italy were invited to participate in a cross-sectional multicentre study; 38 agreed to participate.

The study has been approved by each single “Direzione Sanitaria” (health direction) of all the participating centres. The “Direzione Sanitaria” is the body that must ascertain what should be submitted to the ethical review board or not. The Direzione Sanitaria of the participating centers waived the need for ethical board review for this study.

The study was performed without funding and/or sponsor. The participating centres took part the study voluntarily without change their routine activities on patients. The health personnel on the scene (paramedics, nurses, or doctors) is usually trained to recognize the signs and symptoms of AC according to recommendations issued by the Italian Ministry of Health, including the use of CPSS. Recently, some OCs have started using CPSS during dispatch, but not systematically.

### Study methods

We asked the EMS OCs participating in the study to collect information on all the cases that had been identified by dispatchers with stroke or TIA symptoms and on all the strokes/TIAs identified on the scene by the ambulance health personnel between November 2010 and May 2011 [see Additional file 1: Supplement methods].

Data were collected prospectively through modification of the existing software already in use in the OCs to record dispatches. Data were then collected in an electronic database, through online data entry or electronic file transfer [see Additional file 1: Supplement methods]. Data entry was minimised to avoid any additional work.

The SIS118 coordinating centre checked all the diagnoses reported in the database of each single OC for consistency.

The collection, analysis, and storage were made in anonymous way.

### Outcome and measures

The main outcomes were PPV and sensitivity of stroke/TIA symptoms identification by dispatcher at the OC level.

### Analysis

We computed the PPV and the sensitivity of the dispatchers’ identification of stroke/TIA symptoms. The dispatch was compared to prehospital assessments on the scene. True positives were the cases with stroke/TIA symptoms identified at dispatch and confirmed on the scene, false positives were the cases with stroke/TIA symptoms identified and not confirmed on the scene, false negatives were the cases with stroke/TIA symptoms identified on the scene but not during the dispatch interview.

We performed regression models using the OCs as statistical units. OCs were classified according to the proportion of cases in which the CPSS was performed and reported. The independent variables were PPV, as a function of sensitivity and CPSS use (continuous variable, proportion of cases in which the CPSS score was reported), and sensitivity as a function of PPV and CPSS use. The models were built using generalised linear model command in Stata (v11.0), with identity link, and gaussian residuals and, to take into account other unknown context variables that might have influenced accuracy, we used the regions as cluster. Ninety-five percent confidence intervals (95% CI) are reported for all accuracy estimates and regression coefficients.

## Results

**Figure 1:**
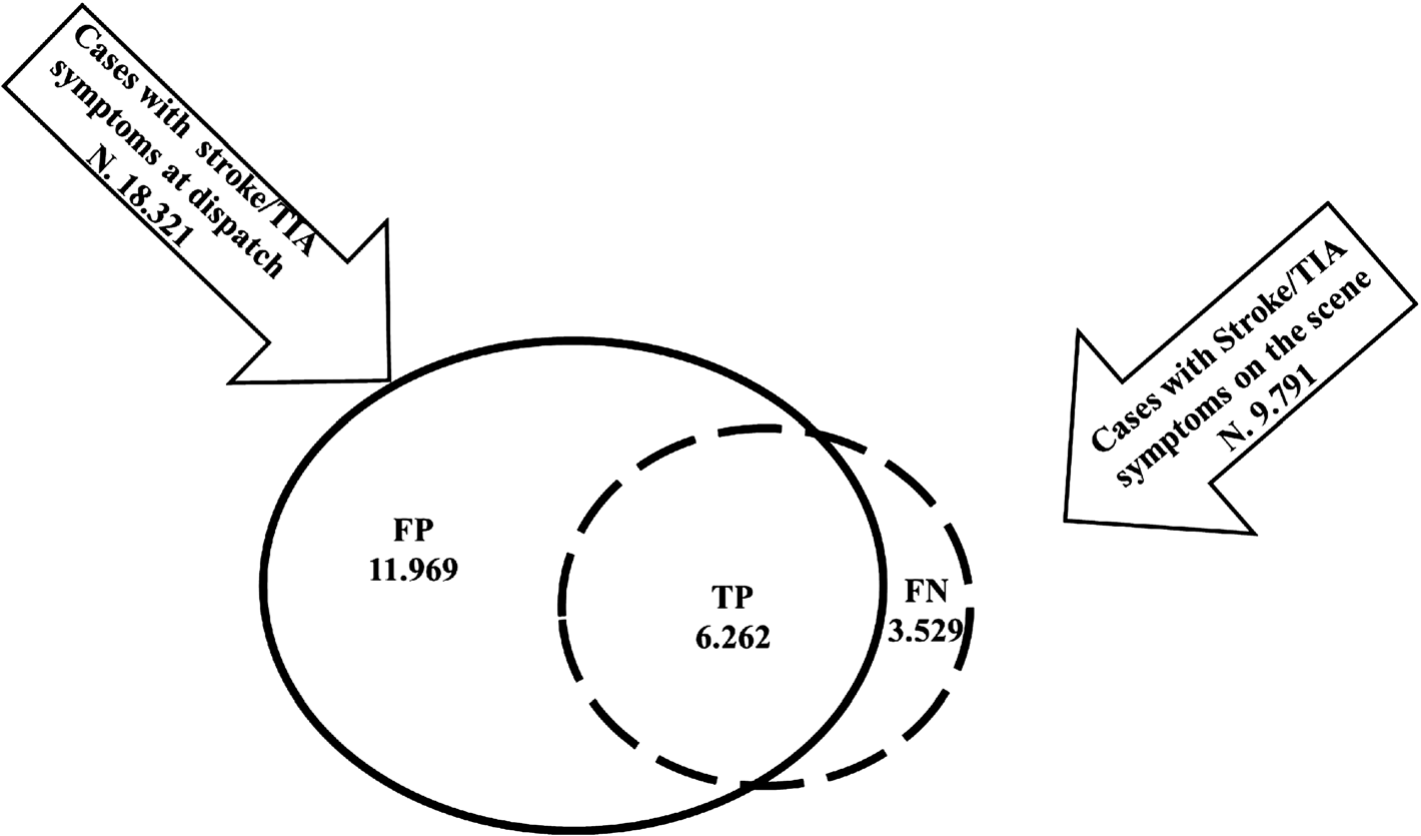
**Description of study population.** Total cases N°21.760.

The study collected data from 38 EMS OCs in 15 of the 20 Italian regions. The participating centres had a reference population of 23 million inhabitants (38% of the Italian population) (please see Additional file 1: Figure S1, Table S3). A total of 21760 cases were included, of which 18231 were the cases with stroke/TIA symptoms at dispatch and 9791 were those identified on the scene (Figure 1). The mean age of the patients with stroke/TIA symptoms confirmed on the scene (n.9791) was 75 years; 53.8% of the patients were female.

During dispatch interviews 79.6% cases of the overall study population (n. 21760) received a high priority code (i.e. red - immediate treatment, or yellow - urgent treatment) at triage (Table 1). We evaluated the concordance between the triage assigned during dispatch and that assigned on the scene. Only 17.2% of the red codes were confirmed (CI 95% 15.9-18.5), while 50% (CI 95% 49.0-50.7) and 40% (CI 95% 37.2-42.7) of yellow and green (treatment can be delayed) triage were confirmed, respectively [see Additional file 1: Table S1]. There were 6262 cases with stroke/TIA symptoms identified at dispatch and confirmed on the scene; thus, positive predictive value of the dispatch was 34.3% (95% CI 33.7-35.0; 6262/18231), while the sensitivity was 64.0% (95% CI 63.0-64.9; 6262/9791). False positives were given a higher priority code at dispatch, while false negatives received a lower priority code (Table 1).

**Table 1:** Cohort characteristics by gender, age, triage, symptoms, use of CPSS

		**Stroke/TIA identification at dispatch**					
	**Total**	**True positive**		**False negative**		**False positive**	
Gender	N	N	%	N	%	N	%
Female	11122	3419	54.6	1848	52.4	5855	48.9
Male	8815	2674	42.7	1547	43.8	4594	38.4
M.I.	1823	169	2.7	134	3.8	1520	12.7
Age							
<=45	2272	411	6.6	99	2.8	1762	14.7
46-55	1225	256	4.1	172	4.9	797	6.7
56-65	1730	529	8.4	286	8.1	915	7.6
66-75	3247	1021	16.3	672	19.0	1554	13.0
76-85	7187	2357	37.6	1384	39.2	3446	28.8
+85	5094	1665	26.6	904	25.6	2525	21.1
M.I.	1005	23	0.4	12	0.3	970	8.1
Triage							
Red	3304	783	12.5	540	15.3	1981	16.6
Yellow	14010	3736	59.7	1564	44.3	8710	72.8
Green	1252	265	4.2	135	3.8	852	7.1
White	32	2	0.0	1	0.0	29	0.2
Not assigned	3162	1476	23.6	1289	36.5	397	3.3
Symptoms collected at dispatch							
Unconscious	2301	739	11.8	452	12.8	1110	9.3
Confusional state	3071	1410	22.5	662	18.8	999	8.3
Not breathing	685	255	4.1	243	6.9	187	1.6
Breathing/conscious	15703	3858	61.6	2172	61.5	9673	80.8
Use of CPSS at dispatch							
CPSS-yes	4976	3038	48.5	449	12.7	1489	12.4
CPSS-no	16784	3224	51.5	3080	87.3	10480	87.6
Total	21760	6262		3529		11969	

CPSS - Cincinnati Prehospital Stroke Scale.

CPSS at dispatch was performed in 22.9% of the cases (Table 1). Four OCs never used CPSS, while 2 OCs used it in 95% of cases. The presence of a CPSS score was positively associated with patient’s age and with symptom-asking at dispatch. The association with triage code was weak, with the exception of a very low percentage in white triages (not urgent/immediate treatment) [see Additional file 1: Table S2].

There was also a strong association between CPSS reporting and the identification of stroke/TIA symptoms at dispatch and the identification of stroke/TIA symptoms on the scene. Both PPV and sensitivity were higher when CPSS was used (Table 2).

**Table 2:** PPV and sensitivity of dispatch for stroke/TIA symptom identification

	**Positive predictive value**			**Sensitivity**		
Age	n/N	%	95% CI	n/N	%	95% CI
<=60	907/4856	18.7	(17.6-19.8)	907/1303	69.6	(67.0-72.1)
>60	5355/13375	40	(39.2-40.9)	5355/8488	63.1	(62.1-64.1)
Presence of CPSS						
Yes	3038/4527	67.1	(65.7-68.5)	3038/3487	87.1	(86.0-88.2)
No	3224/13704	23.5	(22.8-24.2)	3224/6304	51.1	(49.9-52.4)
Centre using CPSS						
> = 10% of cases	4333/7396	58.6	(57.5-59.7)	4333/6098	71.1	(87.0-88.6)
<10% of cases	1929/10835	17.8	(17.1-18.5)	1929/3693	52.2	(50.6-53.9)

CPSS - Cincinnati Prehospital Stroke Scale.

Table 2 presents the PPV and the sensitivity of the dispatch for stroke/TIA by age, presence of CPSS, and centres classified according to CPSS use. For patients over age 60, the PPV was higher and sensitivity was lower than for younger patients.

OCs using the CPSS more often (>10% of cases) had both higher PPV and higher sensitivity.

**Figure 2:**
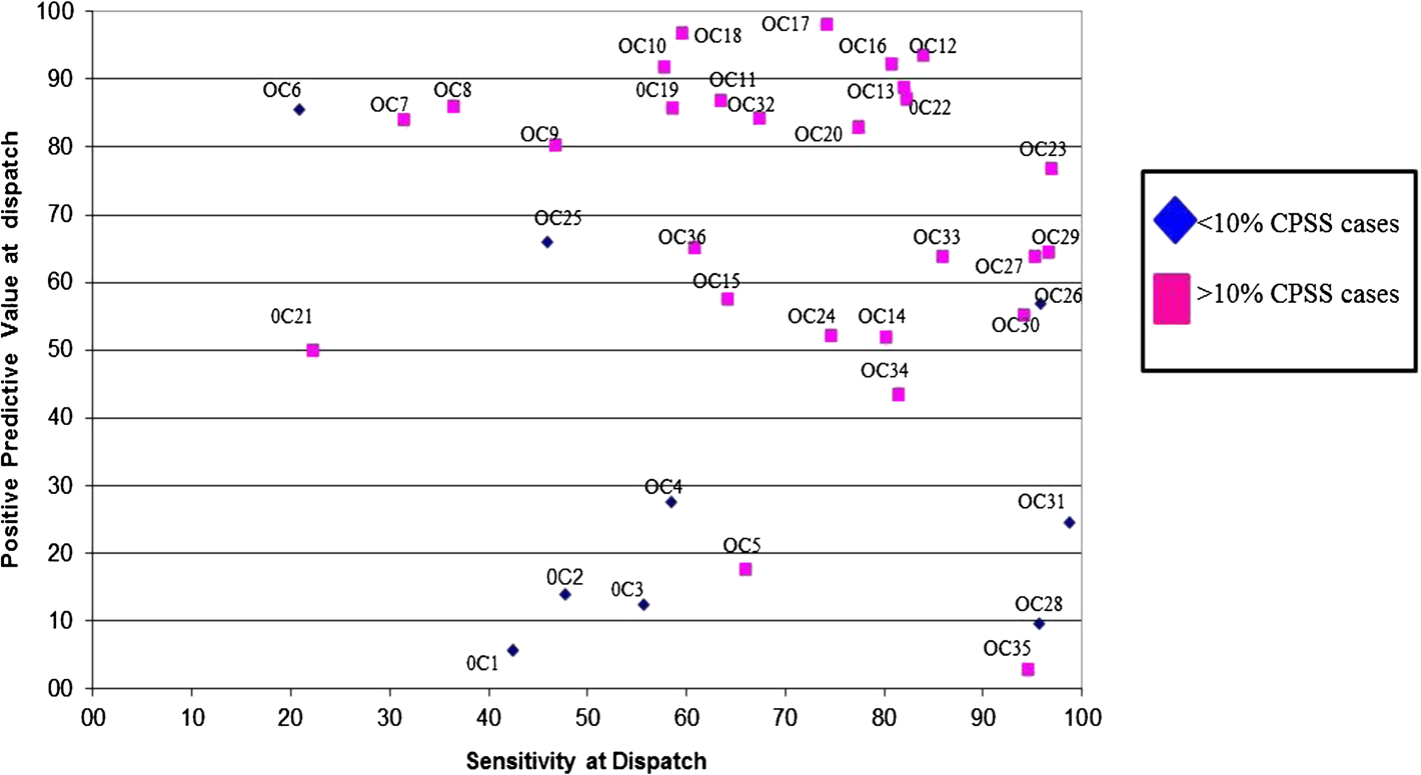
Positive predictive value and sensitivity of participating operative centers.

The graph in Figure 2 plots the PPV and the sensitivity for 36 OCs (two were excluded from the analysis because they had contributed fewer than 20 cases). Diamonds represent the centres that did not use CPSS (<10% of cases) and squares those that did. The former tend to cluster in the area of low PPV and intermediate sensitivity, while the latter tend to cluster in the high PPV and high sensitivity area, with very few exceptions [see Additional file 1: Table S4].

The multivariate analysis (Table 3) shows the association between PPV and sensitivity and between PPV and CPSS use at the OC level. OCs with higher CPSS use tended to have higher PPV and sensitivity even when we adjusted for PPV and sensitivity each other. Centres obtaining a higher PPV tended to have lower sensitivity (borderline statistical significance), and vice versa. Both the models take into account the possible clustering of OCs by regions.

**Table 3:** Linear models

**a. Positive predictive value as function of sensitivity and proportion of cases with CPSS reported**				
	**Coefficient**	Standard error (*)	**p**	**95% CI**
Sensitivity	-0.36	0.192	0.063	-0.73-0.02
Proportion of cases with CPSS	0.48	0.195	0.014	0.10-0.86
Constant	67.11	23.137	0.004	21.76-112.46
**b. Sensitivity as function of positive predictive value and proportion of cases with CPSS reported**				
	**Coefficient**	Standard error (*)	**p**	**95% CI**
Positive predictive value	-0.21	0.138	0.118	-0.48-0.05
Proportion of cases with CPSS	0.32	0.104	0.002	0.12-0.53
Constant	69.32	12.15	0.000	45.50-93.13

(*) Std. Err. adjusted for 14 clusters in regions.

CPSS - Cincinnati Prehospital Stroke Scale.

CPSS use during dispatcher interview was associated with a shorter time interval between call and ambulance referral (mean in minutes: 3.54 vs 4.92) [see Additional file 1: Table S2].

## Discussion

This is the first extensive study on CPSS use by emergency medical dispatchers in Italy. Excluding the two OCs that provided clearly incomplete databases, the study collected consecutive cases of stroke/TIA symptoms both at dispatch and on the scene from about one-third of all the emergency centres in Italy over a six-month period. In Italy, about 170,000 new cases of stroke/TIA are hospitalised every year. Twenty-two percent of cases arrive at the emergency room within 3 hours from symptom onset and only 21% of the witnesses or patients recognize acute stroke/TIA syndrome [31]. Only 45% of stroke/TIA patients arrived at the emergency room by public ambulance [32].

The study expected to include 12,500 cases with stroke/TIA symptoms; as we had nearly 10,000 cases with stroke/TIA symptoms confirmed on the scene, completeness was thus considered acceptable.

Our data showed a strong association between CPSS use and the accuracy of stroke/TIA symptoms identification during the telephone interview: OCs using CPSS were able to correctly identify the stroke/TIA symptoms during phone contact with patient or caller more frequently than were those OCs that use CPSS less frequently; in other words, CPSS use increased dispatch sensitivity. Those OCs using CPSS more frequently also had a lower number of false alarms for patients with stroke/TIA symptoms; in other words, CPSS use increases positive predictive value.

Usually any attempt to improve PPV adversely affects sensitivity, and vice versa*.* This does not seem to be the case for CPSS. We observed the effect both at the individual level and at the OC level. The association at the individual level may be biased for two reasons: 1) because there is surely an association between CPSS use and the suspicion of stroke so that, in those cases in which CPSS was used, a case with stroke symptoms was more likely to be identified; 2) health personnel arrive on the scene already knowing the results of the CPSS at dispatch and this could increase the probability of an agreement in the identification stroke/TIA symptoms. On the other hand, we also observed a strong association at the OC level. In this analysis, a bias is very unlikely given that as we selected all the cases with stroke symptoms at dispatch and all the stroke symptoms identified on the scene, the OCs were classified according to the proportion of cases in which CPSS was reported among all the cases, even the false negative. The accuracy of stroke/TIA symptoms identification was thus attributed as a characteristic of the OCs according to the performance obtained on the whole population, with and without CPSS.

Other studies have tried to measure the effect of several operational scales or protocols on the accuracy of dispatch stroke and TIA identification [14,18,19,23]. The sensitivity and PPV range between 42% and 85% in Ramanujam’s study, between 41% and 45% in Buck’s study, and between 47.6% and 49% in Deakin’s study, where the protocol used (Medical Priority Dispatch System) did not contain queries on motor stroke symptoms and where the stroke/TIA diagnosis upon discharge from the Emergency Department was the reference standard. None of the above-mentioned studies used as reference or gold standard the identification of stroke symptoms on the scene.

Our study shows an extreme variability in the performance among OCs (Figure 2). This variability is only partially explained by the use of CPSS. Furthermore, even when adjusting for CPSS use, the association between PPV and sensitivity is very weak. If the centres were operating under close to optimal conditions, any advantage in PPV would have a negative impact on sensitivity and vice versa. We can deduce that many centres are operating under far from optimal conditions and are not working on the theoretical frontier of the sensitivity and specificity trade-off. This could be due to many organizational factors as well as to problems related to operator training.

Not surprisingly, dispatch had a higher PPV and lower sensitivity for people over 60. Less attention and a stricter application of urgency criteria for older people have been observed in injured patients [33].

### Limits

In this observational study we cannot rule out the possibility that the association between CPSS use and accuracy in identifying stroke/TIA symptoms at dispatch may have been confounded by other factors. In particular, there could be organisational components influencing both CPSS use and emergency OC performance: better-organised centres may be more likely to use CPSS and, for other reasons, may be more skilled at identifying stroke. This is in line with the observation that OCs using CPSS also collected information on symptoms at dispatch more often. In order to control for this possible bias, the multivariate models took into account the homogeneity among OCs of the same region; this statistical method should partially adjust for other unknown organisational factors acting at the regional level, i.e. the administrative organization having responsibility for health care organization in Italy. Furthermore, the conduction of the survey itself, with its data collection systems, may have increased the use of CPSS in some OCs; how this could have affect the relation between CPSS use and stroke/TIA symptom identification is not predictable.

Another limit of the study is that we used stroke/TIA identification symptoms on the scene as our gold standard; we do not know how many of these cases had a confirmed diagnosis of stroke or TIA at the end of hospitalization. Unfortunately there is no mean to confirm the diagnosis in our study. Nevertheless, the concordance in stroke/TIA symptom recognition between the two prehospital emergency steps (dispatch and on the scene) has intrinsic value: from an operational point of view, a case not classified as possible stroke at dispatch but treated as one on the scene causes the same logistical problems (i.e., not having the right ambulance or not having pre-alerted the stroke unit), regardless of whether or not stroke is confirmed at the end of the diagnostic process. On the other hand, a case identified with stroke symptoms at dispatch but not confirmed on the scene and treated as another disorder will result in a waste of resources, regardless of whether or not the final diagnosis is indeed cerebrovascular disease.

The Italian Ministry of Health issued guidelines on the management of AC patients in prehospital emergency service, including CPSS use. In Italy, the health personnel on the ambulances are trained to use CPSS to recognize AC; at the moment the same training is not required for dispatchers, but, according to our results, this is a critical point and training requirements for dispatchers should be reviewed.

## Conclusions

Centres that use CPSS more frequently during phone dispatch showed greater agreement with on-the-scene prehospital assessments, both in correctly identifying more stroke/TIA symptoms and in giving fewer false positives for non-stroke/TIA cases. Our study shows an extreme variability in the performance among OCs, highlighting that form many centres there is room for improvement in both sensitivity and positive predictive value of the dispatch. Our results should be used for benchmarking proposals in the effort to identify best practices across the country.

## Abbreviations

CPSS: Cincinnati Prehospital Stroke Scale; TIA: Transient ischemic attack; EMS: Emergency medical service; PPV: Positive predictive value; AC: Acute cerebrovascular disease; OC: Operative center; ED: Emergency Department; 95% CI: Ninety-five percent confidence intervals.

## Competing interests

The authors declare that they have no competing interests. The authors alone are responsible for the content and writing of the paper.

## Authors’ contributions

AD,GFV, PGR conceived the study and designed the study. AD, GFV supervised the conduct of the study and data collection, undertook recruitment of participating centers and managed the data, including quality control. PGR provided statistical advice on study design and analyzed the data. AD, PGR drafted the manuscript. AD,GFV, PGR contributed substantially to its revision. AD takes responsibility for the paper as a whole. All authors read and approved the final manuscript.

## Authors’ information

Italian Prehospital Management of Acute Stroke Group of the Italian Society of prehospital emergency Services (SIS118) – Coordinators: Guido Francesco Villa (Lc), Fulvio Bussani (past President of SIS118), Fedele Clemente (President of SIS118) (Cb), Assunta De Luca (Rm), Alessandro Caminiti (Rm), Maurizio Moroni (Rm), Andrea Pagliosa (Mi), Francesco Bermano (Ge), Marilena Campisi (Mo), Stefano Ferlito (Im), Claudio Martina (Bi); Riccardo Sestili (An), Sabrina Toppi (An),. All members of group contribute to conceive the study and to supervised the conduct of the study.

## Pre-publication history

The pre-publication history for this paper can be accessed here:

http://www.biomedcentral.com/1472-6963/13/513/prepub

## Supplementary Material

Additional file 1 The use of Cincinnati Prehospital Stroke Scale during telephone dispatch interview increases the accuracy in identifying stroke and transient ischemic attack symptoms. Click here for file

## Support for this study

The Regional Authority of Emergency Services (ARES 118) Lazio Region for creating the electronic data sheet and the specific website to permit participating Operative Centers to enter data.
