# Supplementary material for: The use of Cincinnati prehospital stroke scale during telephone dispatch interview increases the accuracy in identifying stroke and transient ischemic attack symptoms
Source: BMC Health Serv Res. 2013 Dec 11;13:513. doi: 10.1186/1472-6963-13-513 (PMC3867422; doi:10.1186/1472-6963-13-513)
Supplement: Additional file 1 — The use of Cincinnati Prehospital Stroke Scale during telephone dispatch interview increases the accuracy in identifying stroke and transient ischemic attack symptoms. [file 1472-6963-13-513-S1.doc]

**ADDITIONAL DOCUMENTATION**

**The use of Cincinnati Prehospital Stroke Scale during telephone dispatch interview increases the accuracy in identifying stroke and transient ischemic attack symptoms**

Assunta De Luca, Paolo Giorgi Rossi, Guido Francesco Villa and the Italian Group Pre-hospital management of acute stroke – Italian Society of pre hospital emergency Services (SIS118) .

**SUPPLEMENT METHODS**

| **List of variables in the electronic database for the online data entry** |
| --- |
| **Personal information** |
| Operative center (OC) code |
| Province and region of OC |
| Time of call |
| Address of event |
| Gender |
| Sex |
| **Information at dispatch** |
| Status of consciousness |
| Status of breathing |
| CPSS (facial droop, arm drift, speech)* |
| Sudden onset strong headache |
| Vomit |
| Time symptoms onset |
| Suspected diagnosis (cerebrovascular diseases; other pathologies) |
| Code triage on call (Red, yellow, green, white)** |
| Time ambulance activation |
| Type of activated ambulance |
| **Information on the scene** |
| Vital parameters |
| CPSS (facial droop, arm drift, speech) |
| Other symptoms |
| Time symptoms onset |
| Diagnosis (stroke or transient ischemic attacks) |
| Type of treatment on the scene |
| Code Triage on the scene (Red, yellow, green, white) ** |
| Time arrival on the scene |
| Type of referral hospital (hub or spoke) |

**The questions on signs are asked to relatives or laypersons by dispatchers.*

***Triage code: red (immediate treatment and transportation); yellow (urgent treatment and transportation) green (treatment and transportation can be delayed); white (not needed EMS treatment and transportation)*

**List of the Prehospital Emergency Medical Service (EMS) Operative Centers (OCs) participating in the study.**

The Directors of OCs authorised their centres to take part in the study and did everything necessary to make the study possible. The Directors of OCs designated the local Study Coordinators. The OCs collected information prospectively on all the cases that had been identified by dispatchers with stroke or TIA symptoms and all the stroke/TIA symptoms identified on the scene by the ambulance health personnel**.** In particular, the CPSS data are usually reported on *ad hoc* electronic clinical sheet by dispatcher and health personnel on the scene

| **Italian Regions** | | **EMS Operative Centers (OC)** | **City** | | **Director of OC (participating investigators)** | | **Local coordinators of Study** | |
| --- | --- | --- | --- | --- | --- | --- | --- | --- |
| BASILICATA | | 118 Basilicata | Potenza. Matera | | Dr. Libero Mileti | | Dr. Serafino Rizzo; Giuseppe Lorusso | |
| CALABRIA | | Suem 118 Crotone | Crotone | | Dr. Gaspare Muraca | | Dr. Giuseppina Masceri | |
| EMILIA ROMAGNA | | Modena Soccorso 118 | Modena | | Dr. Marilena Campisi | | Dr Alessandra Silvestri | |
| EMILIA ROMAGNA | | Bologna Soccorso 118 | Bologna | | Dr. Giovanni Gordini | | Dr. Maurizio Liberti | |
| FRIULI-VG | | Regione Soccorso118 | Trieste | | Dr. Vittorio Antonaglia | |  | |
| LAZIO | | Regional Authority of Emergency Services ARES118 |  | | Dr. Antonio De Santis | | Dr. Assunta De Luca. Maurizio Moroni. Marilisa Carbonari. | |
| LAZIO | | Frosinone | Frosinone | | Dr. Lauro Sciannamea | | Dr. Antonietta Cattani | |
| LAZIO | | Latina | Latina | | Dr. Paolo Viola | | Dr. Claudio De Santis | |
| LAZIO | | Rieti | Rieti | | Dr. Alfonso Tesoriere | | Dr. Bruno Colio | |
| LAZIO | | Roma capitale | Roma | | Dr. Livio De Angelis | | Nadia La Trofa | |
| LAZIO | | Roma provincia | Roma prov | | Dr. Anna Maria Matarese | | Dr. Alessandro Caminiti | |
| LAZIO | | Viterbo | Viterbo | | Dr Vittorio Altomani | | Dr. Claudio Quintarelli | |
| LIGURIA | | 118 Savona | Savona | | Dr. Salvatore Esposito | | Dr. Lucia Testa | |
| LIGURIA | | Imperia Soccorso118 | Imperia | | Dr. Ferlito Stefano | | Dr. Annalisa Belluti; Dr. Pierangelo Ferrari | |
| LOMBARDIA | | Regional Authority of Emergency Urgency AREU |  | | Dr. Alberto. Zoli | | Dr. Andrea Pagliosa. Dr. Marco Salmoiraghi. Dr. Stefania Favretti. | |
| LOMBARDIA | | 118 Bergamo | Bergamo | | Dr. Oliviero Valoti | |  | |
| LOMBARDIA | | 118 Brescia | Brescia | | Dr. Claudio Mare | |  | |
| LOMBARDIA | | 118 Cremona | Cremona | | Dr. Romano Paolucci | |  | |
| LOMBARDIA | | 118 Lecco | Lecco | | Dr. Guido Francesco Villa | |  | |
| LOMBARDIA | | 118 Mantova | Mantova | | Dr. Gian Paolo Castelli | |  | |
| LOMBARDIA | | 118 Brianza | Monza MI | | Dr. Gian Piera Rossi | |  | |
| LOMBARDIA | | 118 Como | Como | | Dr. Mario Landriscina | |  | |
| LOMBARDIA | 118 Pavia | | | Pavia | | Dr. Maurizio Raimondi | |  |
| LOMBARDIA | 118 Soccorso Sanitario MI | | | Milano | | Dr. Giovanni Sesana | |  |
| LOMBARDIA | 118 Sondrio | | | Sondrio | | Dr. Paolo Della Torre | |  |
| LOMBARDIA | 118 Varese | | | Varese | | Dr. Guido Garzena | |  |
| MARCHE | Ancona Soccorso 118 | | | Ancona | | Dr. Riccardo Sestili | | Dr. Sabrina Toppi |
| MOLISE | 118 Molise Soccorso | | | Campobasso | | Dr. Fedele Clemente | | Dr. Vincenzo Di Iorio |
| PIEMONTE | 118 Novara | | | Novara | | Dr. Egle Maria Valle | | Dr. Salvatore Izzo |
| PIEMONTE | C.O. 118 e Base elisoccorso | | | Alessandria | | Dr. Giovanni Lombardi | |  |
| PIEMONTE | Emergenza Sanitaria118 | | | biella | | Dr. Claudio Martina | | Dr. Erika Aimino. Alberto Tirapelle |
| PIEMONTE | Chirurgia generale CTO | | | Torino + Grugliasco | | Dr. Francesco Enrichens | |  |
| PIEMONTE | Cuneo 118 | | | Cuneo + Saluzzo | | Dr. Luigi Silimbri | | Dr. Stefano Quaranta |
| PIEMONTE | Emergenza Sanitaria118 | | | Asti | | Dr. Roberto Balagna | | Dr. Francesco Fiorillo |
| PIEMONTE | Emergenza Sanitaria118 | | | Premosello VVB | | Dr. Paolo Gramatica | |  |
| PIEMONTE | Emergenza Sanitaria118 | | | Borgosesia VC | | Dr. Giuseppe Cannata | |  |
| PUGLIA | 118 Puglia | | | Bari | | Dr. Gaetano Di Pietro | | Dr. Cesare Calamita |
| SARDEGNA | 118 Sassari | | | Sassari | | Dr. Piero De Logu | | Dr. Alessandra Meloni |
| TOSCANA | 118 Siena | | | Siena | | Dr. Francesco Palumbo | | Dr. Stefano Dami |
| TOSCANA | 119 Massa Carrara Soccorso | | | Massa Capo dip. | | Dr. Roberto Vatteroni | |  |
| TOSCANA | CO Empoli | | | Empoli | | Dr. Stefano Pappagallo | | Dr. Cecilia Marmai; Dr. Lubrani Alessio |
| TOSCANA | 118 Pistoia | | | Pistoia | | Dr. Piero Paolini | | Dr. Roberto Mariani |
| TOSCANA | 118 Pisa | | | Pisa | | Dr. Paolo Tognarelli | |  |
| UMBRIA | Umbria Soccorso | | | Perugia | | Dr. Mario Capruzzi | | Dr. Giocondo Bocciarelli |
| VENETO | 118 Rovigo | | | Rovigo | | Dr. Marco Sommacampagna | | Dr. Luca Guerra |

**SUPPLEMENTAL FIGURE – RESULTS**

**Figure S1. Prehospital Emergency Medical Service (EMS) Operative Centres Participating in the Study**


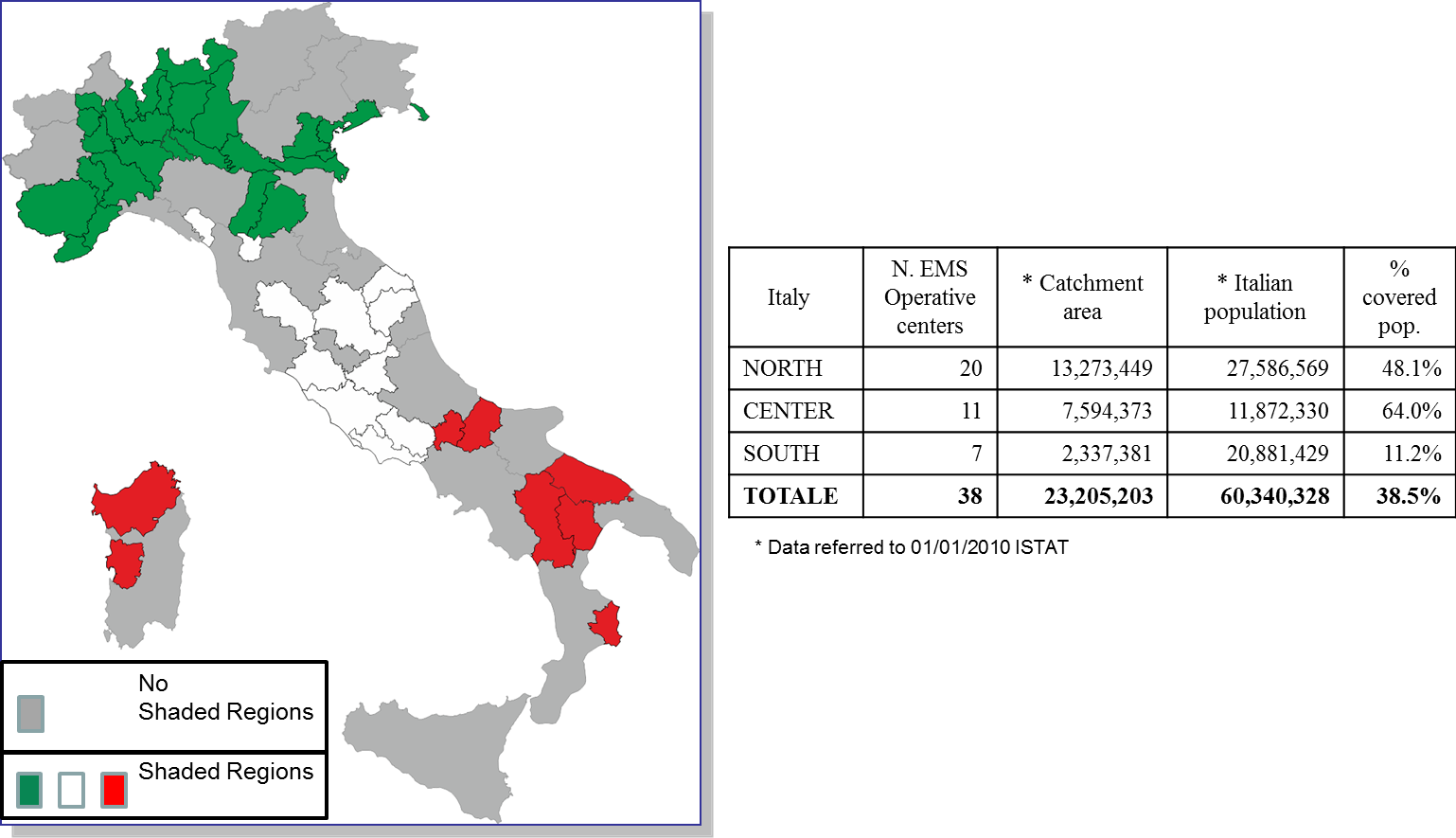


**SUPPLEMENTAL TABLES – RESULTS**

**Table S1. Concordance of Triage codes assigned during dispatch and on site**

|  | **Dispatch triage** | | | |  |
| --- | --- | --- | --- | --- | --- |
| **On-site triage** | Red | Yellow | Green | White | total |
|  | n | n | n | n | N |
| dead | 80 | 42 | 0 | 0 | 122 |
| Red | 568 | 776 | 11 | 2 | 1357 |
| Yellow | 1440 | 6983 | 182 | 3 | 8608 |
| Green | 393 | 2257 | 500 | 4 | 3154 |
| White | 224 | 1115 | 105 | 2 | 1446 |
| Total | 2705 | 11173 | 798 | 11 | 14687 |
| *Proportion confirmed* | *17.2* | *49.8* | *39.9* | *6.3* |  |
| *95% CI* | *15.9-18.5* | *49.0-50.7* | *37.2-42.7* | *0.8-20.1* |  |
| Kappa= 0.148 |  |  |  |  |  |

**Table S2. Presence of CPSS score at dispatch by gender, age, triage, symptoms of stroke/TIA**

|  |  |  |  | Presence of CPSS score at dispatch | | | | |
| --- | --- | --- | --- | --- | --- | --- | --- | --- |
|  |  | total |  | yes |  |  | no |  |
| Gender |  | N |  | N | % |  | N | % |
| female |  | 11122 |  | 2766 | 24.9 |  | 8356 | 38.4 |
| male |  | 8815 |  | 2129 | 24.2 |  | 6686 | 30.7 |
| m.i. |  | 1823 |  | 81 | 4.4 |  | 1742 | 8.0 |
|  |  |  |  |  |  |  |  |  |
| Age |  |  |  |  |  |  |  |  |
| <=45 |  | 2272 |  | 296 | 13.0 |  | 1976 | 9.1 |
| 46-55 |  | 1225 |  | 202 | 16.5 |  | 1023 | 4.7 |
| 56-65 |  | 1730 |  | 440 | 25.4 |  | 1290 | 5.9 |
| 66-75 |  | 3247 |  | 837 | 25.8 |  | 2410 | 11.1 |
| 76-85 |  | 7187 |  | 1924 | 26.8 |  | 5263 | 24.2 |
| +85 |  | 5094 |  | 1262 | 24.8 |  | 3832 | 17.6 |
| m.i. |  | 1005 |  | 15 | 1.5 |  | 990 | 4.5 |
|  |  |  |  |  |  |  |  |  |
| Triage |  |  |  |  |  |  |  |  |
| Red |  | 3304 |  | 613 | 18.6 |  | 2691 | 12.4 |
| Yellow |  | 14010 |  | 2940 | 21.0 |  | 11070 | 50.9 |
| Green |  | 1252 |  | 248 | 19.8 |  | 1004 | 4.6 |
| White |  | 32 |  | 2 | 6.3 |  | 30 | 0.1 |
| not assigned |  | 3162 |  | 1173 | 37.1 |  | 1989 | 9.1 |
|  |  |  |  |  |  |  |  |  |
| Symptoms collected at dispatch |  |  |  |  |  |  |  |  |
| unconscious |  | 2301 |  | 657 | 28.6 |  | 1644 | 7.6 |
| confusional state |  | 3071 |  | 1019 | 33.2 |  | 2052 | 9.4 |
| not breathing |  | 7467 |  | 588 | 7.9 |  | 97 | 0.4 |
| breathing/conscious |  | 8921 |  | 2712 | 30.4 |  | 12991 | 59.7 |
|  |  |  |  |  |  |  |  |  |
| Stroke/TIA symptoms at dispatch |  |  |  |  |  |  |  |  |
| true positive |  | 6262 |  | 3038 | 48.5 |  | 3224 | 14.8 |
| false positive |  | 11969 |  | 1489 | 12.4 |  | 10480 | 48.2 |
| false negative |  | 3529 |  | 449 | 12.7 |  | 3080 | 14.2 |
|  |  |  |  |  |  |  |  |  |
| Time from call to ambulance start (minutes) |  |  |  |  |  |  |  |  |
| mean |  | 4.29 |  | 3.54 | - |  | 4.92 | - |
| standard deviation |  | 4.27 |  | 4.27 | - |  | 7.58 | - |
|  |  |  |  |  |  |  |  |  |
| Total |  | 21760 |  | 4976 | 22.9 |  | 16784 | 77.1 |

CPSS - Cincinnati Prehospital Stroke Scale

**Table S3. Centre characteristics and characteristics of patients, according to centre use of CPSS at dispatch.**

|  |  |  |  | **Use of CPSS at dispatch** | | | | |
| --- | --- | --- | --- | --- | --- | --- | --- | --- |
|  |  | **total** |  | **<10% of cases** | |  | **>=10% of cases** | |
|  |  | **N** |  | **N** | **%** |  | **N** | **%** |
| Number of centres |  |  |  |  |  |  |  |  |
|  |  | 38 |  | 10 | 26.3* |  | 28 | 73.7* |
| Geographic area |  |  |  |  |  |  |  |  |
| North |  | 20 |  | 3 | 30.0 |  | 17 | 60.7 |
| Centre |  | 11 |  | 5 | 50.0 |  | 6 | 21.5 |
| South and Island |  | 7 |  | 2 | 20.0 |  | 5 | 17.8 |
|  |  |  |  |  |  |  |  |  |
| Total number of patients | | | |  |  |  |  |  |
|  |  | 21760 |  | 12599 | 57.9* |  | 9161 | 42.1* |
|  |  |  |  |  |  |  |  |  |
| Gender |  |  |  |  |  |  |  |  |
| female |  | 11122 |  | 6104 | 48.4 |  | 5018 | 54.8 |
| male |  | 8815 |  | 4917 | 39.0 |  | 3898 | 42.5 |
| m.i. |  | 1823 |  | 1578 | 12.5 |  | 245 | 2.7 |
|  |  |  |  |  |  |  |  |  |
| Age |  |  |  |  |  |  |  |  |
| <=45 |  | 2272 |  | 1645 | 13.1 |  | 627 | 6.8 |
| 46-55 |  | 1225 |  | 799 | 6.3 |  | 426 | 4.7 |
| 56-65 |  | 1730 |  | 914 | 7.3 |  | 816 | 8.9 |
| 66-75 |  | 3247 |  | 1764 | 14.0 |  | 1483 | 16.2 |
| 76-85 |  | 7187 |  | 3750 | 29.8 |  | 3437 | 37.5 |
| +85 |  | 5094 |  | 2782 | 22.1 |  | 2312 | 25.2 |
| m.i. |  | 1005 |  | 945 | 7.5 |  | 60 | 0.7 |
|  |  |  |  |  |  |  |  |  |
| Triage |  |  |  |  |  |  |  |  |
| Red |  | 3304 |  | 1973 | 15.7 |  | 1331 | 14.5 |
| Yellow |  | 14010 |  | 9163 | 72.7 |  | 4847 | 52.9 |
| Green |  | 1252 |  | 879 | 7.0 |  | 373 | 4.1 |
| White |  | 32 |  | 26 | 0.2 |  | 6 | 0.1 |
| not assigned |  | 3162 |  | 558 | 4.4 |  | 2604 | 28.4 |
|  |  |  |  |  |  |  |  |  |
| Symptoms collected at dispatch | | |  |  |  |  |  |  |
| unconscious |  | 2301 |  | 1283 | 10.2 |  | 1018 | 11.1 |
| confusional state |  | 3071 |  | 1606 | 12.7 |  | 1465 | 16.0 |
| not breathing |  | 685 |  | 379 | 3.0 |  | 306 | 3.3 |
| breathing/conscious |  | 15703 |  | 9331 | 74.1 |  | 6372 | 69.6 |
|  |  |  |  |  |  |  |  |  |
| Stroke/TIA symptoms confirmed on site | | |  |  |  |  |  |  |
| yes |  | 11969 |  | 3693 | 29.3 |  | 6098 | 66.6 |
| no |  | 9791 |  | 8906 | 70.7 |  | 3063 | 33.4 |

CPSS - Cincinnati Prehospital Stroke Scale ***;***** Row percentage.*

**Table S4. Operative Centres (OCs) sensitivity and PPV.**

| **OCs*** | **Geographic area** | **Sensitivity** | **PPV** | **>10% CPSS YES/N0** |
| --- | --- | --- | --- | --- |
| OC28 | Centre | 95.59 | 9.73 | no |
| OC4 | Centre | 58.43 | 27.59 | no |
| OC3 | Centre | 55.66 | 12.50 | no |
| OC2 | Centre | 47.76 | 13.91 | no |
| OC1 | Centre | 42.37 | 5.67 | no |
| OC31 | North | 98.73 | 24.53 | no |
| OC26 | North | 95.81 | 56.84 | no |
| OC6 | North | 20.84 | 85.48 | no |
| OC25 | South & Island | 45.89 | 65.95 | no |
| OC29 | Centre | 96.61 | 64.41 | yes |
| OC27 | Centre | 95.21 | 63.93 | yes |
| OC30 | Centre | 94.05 | 55.24 | yes |
| OC33 | Centre | 85.86 | 63.81 | yes |
| OC16 | Centre | 80.77 | 92.31 | yes |
| OC5 | Centre | 65.99 | 17.60 | yes |
| OC23 | North | 96.85 | 76.88 | yes |
| OC35 | North | 94.59 | 2.86 | yes |
| OC12 | North | 83.94 | 93.58 | yes |
| OC22 | North | 82.27 | 86.98 | yes |
| OC13 | North | 82.06 | 88.84 | yes |
| OC34 | North | 81.48 | 43.35 | yes |
| OC14 | North | 80.13 | 51.93 | yes |
| OC24 | North | 74.65 | 52.22 | yes |
| OC32 | North | 67.37 | 84.21 | yes |
| OC11 | North | 63.44 | 86.75 | yes |
| OC36 | North | 60.88 | 65.18 | yes |
| OC10 | North | 57.69 | 91.84 | yes |
| OC15 | North | 57.50 | 53.49 | yes |
| OC9 | North | 48.67 | 77.11 | yes |
| OC8 | North | 36.48 | 85.86 | yes |
| OC7 | North | 31.47 | 83.91 | yes |
| OC21 | North | 22.22 | 50.00 | yes |
| OC20 | South & Island | 77.36 | 82.83 | yes |
| OC17 | South & Island | 74.24 | 98.00 | yes |
| OC18 | South & Island | 59.62 | 96.88 | yes |
| OC19 | South & Island | 58.62 | 85.71 | yes |
| ** OCs reported in the figure 2 of manuscript.* | | | |  |
